# Leveraging DNA Damage Response Signaling to Identify Yeast Genes Controlling Genome Stability

**DOI:** 10.1534/g3.115.016576

**Published:** 2015-02-26

**Authors:** Jason A. Hendry, Guihong Tan, Jiongwen Ou, Charles Boone, Grant W. Brown

**Affiliations:** * Department of Biochemistry, University of Toronto, Toronto, Ontario, Canada; † Donnelly Centre, University of Toronto, Toronto, Ontario, Canada; ‡ Department of Molecular Genetics, University of Toronto, Toronto, Ontario, Canada

**Keywords:** ribonucleotide reductase, DNA repair, DNA damage, DNA replication, genome instability

## Abstract

Oncogenesis frequently is accompanied by rampant genome instability, which fuels genetic heterogeneity and resistance to targeted cancer therapy. We have developed an approach that allows precise, quantitative measurement of genome instability in high-throughput format in the *Saccharomyces cerevisiae* model system. Our approach takes advantage of the strongly DNA damage-inducible gene *RNR3*, in conjunction with the reporter synthetic genetic array methodology, to infer mutants exhibiting genome instability by assaying for increased Rnr3 abundance. We screen for genome instability across a set of ~1000 essential and ~4200 nonessential mutant yeast alleles in untreated conditions and in the presence of the DNA-damaging agent methylmethane sulfonate. Our results provide broad insights into the cellular processes and pathways required for genome maintenance. Through comparison with existing genome instability screens, we isolated 130 genes that had not previously been linked to genome maintenance, 51% of which have human homologs. Several of these homologs are associated with a genome instability phenotype in human cells or are causally mutated in cancer. A comprehensive understanding of the processes required to prevent genome instability will facilitate a better understanding of its sources in oncogenesis.

Genome maintenance is critical for cell viability and frequently is compromised in cancer. Loss of genome stability can be caused by exposure to sources of DNA damage, or defects in DNA repair or DNA replication genes. Genome instability can result in a diverse set of effects on DNA, including point mutations, small insertions and deletions, chromosome translocation or truncation, loss of heterozygosity, and even changes in ploidy (reviewed in Aguilera and Gomez-Gonzalez 2008; Storchova and Pellman 2004). Cancer cells frequently suffer from increased mutation rates and abnormal karyotypes, indicative of genome instability (reviewed in Vogelstein *et al.* 2013). In fact, genome instability underlies the genetic heterogeneity that stymies targeted cancer therapy (Glickman and Sawyers 2012; Vogelstein *et al.* 2013). Understanding the set of genes that function to maintain genome stability is critical to address the sources of genome instability in cancer. In somatic cancers especially, these sources remain unclear (Negrini *et al.* 2010).

Several genome-wide screens assaying genome instability have been conducted with the budding yeast *S. cerevisiae* (Alvaro *et al.* 2007; Alver *et al.* 2013; Andersen *et al.* 2008; Askree *et al.* 2004; Cheng *et al.* 2012; Huang *et al.* 2003; Stirling *et al.* 2011; Smith *et al.* 2004; Yu *et al.* 2006). Because genome instability has diverse consequences, these screens have used a wide array of assays. Many focus on the consequences of genome instability on DNA, by assaying loss of genetic markers, increased mutation rate, or changes in telomere length (Andersen *et al.* 2008; Askree *et al.* 2004; Huang *et al.* 2003; Stirling *et al.* 2011). Others focus on assaying the relocalization of DNA repair or DNA damage checkpoint proteins to nuclear foci, which indicates their active involvement in genome maintenance (Alvaro *et al.* 2007; Cheng *et al.* 2012). Another approach is to assay for activation of the DNA damage response (DDR) pathway, a signaling pathway that coordinates DNA repair processes with the cell-cycle (reviewed in Harrison and Haber 2006; Putnam *et al.* 2009). The DDR causes strong transcriptional induction of the gene *RNR3*, and assaying Rnr3 abundance has been used to identify genome maintenance genes (Zhou and Elledge 1992). However, the Rnr3 abundance screen has yet to be combined with modern reagents and methodology for yeast genetics, such as the nonessential deletion mutant collection (Giaever *et al.* 2002), and synthetic genetic array (SGA) technology (Tong and Boone 2006).

In *S. cerevisiae*, activation of the DDR begins when the single-stranded binding protein, RPA, coats exposed tracts of single-stranded DNA (ssDNA). Next, ssDNA-RPA is bound by Ddc2, in complex with the large phosphatidylinositol 3′ kinase-like kinase Mec1 (Rouse and Jackson 2002; Zou and Elledge 2003). Mec1 is activated subsequently through interactions with multiple nearby protein assemblies, the most prominent being the 9-1-1 (Mec3-Rad17-Ddc1) clamp (Majka *et al.* 2006; Navadgi-Patil and Burgers 2009). Another large phosphatidylinositol 3′ kinase-like kinase, Tel1, is uniquely recruited to and activated at double-stand breaks (Falck *et al.* 2005; Nakada *et al.* 2003). Activated Mec1 and Tel1 propagate the damage signal by hyperphosphorylating the signaling adaptor Rad9, which mediates phosphorylation of the transducing kinase Rad53 (Naiki *et al.* 2004; Schwartz *et al.* 2002; Sweeney *et al.* 2005). Rad53 amplifies the DNA damage signaling through extensive autophosphorylation (Sun *et al.* 1998). In addition, Rad53 phosphorylates downstream targets, communicating the presence of DNA damage to diverse cellular processes. Two well-understood effects of Rad53 activation are the inhibition of late origin firing during S-phase (Zegerman and Diffley 2010; Lopez-Mosqueda *et al.* 2010) and prevention of sister chromatid separation in anaphase (Sanchez *et al.* 1999; Agarwal *et al.* 2003), both of which allow time for DNA repair to occur.

Another well-studied effect of Rad53 activation is the transcriptional induction of the ribonucleotide reductase (RNR) complex, which catalyzes the rate-limiting step in dNTP synthesis. Many repair processes involve a DNA synthesis step and increased dNTP levels promote survival after DNA damage (Chabes *et al.* 2003). In *S. cerevisiae*, the RNR complex is encoded by two small (*RNR2* and *RNR4*) and two large subunits (*RNR1* and *RNR3*) (reviewed in Sanvisens *et al.* 2013). In a normal cell cycle, two Rnr1 subunits associate with Rnr2 and Rnr4 in an α_2_ββ’ architecture, with Rnr3 abundance being negligible. DDR activation induces the transcription of all four *RNR* subunits, albeit by different mechanisms and to different extents. *RNR2*, *RNR3* and *RNR4* contain 13bp damage response elements (DREs) in their promoters, which are bound by the transcriptional repressor Crt1/Rfx1 (Huang *et al.* 1998). Active Rad53 induces transcription of these *RNR* genes by phosphorylating the kinase Dun1, which in turn hyperphosphorylates Crt1, causing its dissociation from DNA (Huang *et al.* 1998). *RNR1* lacks DREs in its promoter and is induced in a Mec1-Rad53−dependent but Dun1-independent manner that involves a different transcription factor, Ixr1 (Tsaponina *et al.* 2011).

Rnr3 is an ideal read-out of DDR pathway activation, and by extension genome instability. First, it is a well-characterized transcriptional target of the DNA damage response (Huang *et al.* 1998). *RNR3* up-regulation in response to exogenous DNA-damaging agents like methylmethane sulfonate (MMS) has been demonstrated at both the mRNA and protein level and depends on known DDR kinases (Huang *et al.* 1998; Li and Reese 2001; Tsaponina *et al.* 2011). Second, mutation of several well-characterized DNA repair and replication genes leads to constitutive expression of *RNR3*, demonstrating that *RNR3* can be induced by both exogenous (*i.e.*, environmental) and endogenous (*i.e.*, genetic perturbation) sources of genome instability (Tang *et al.* 2009; Davidson *et al.* 2012). Finally, expression of *RNR3* is negligible in the absence of perturbation, but it is precipitously induced in response to DNA damage, by far the greatest induction of all the *RNR* genes (Elledge *et al.* 1993). The original Constitutive RNR Three (CRT) screen used an *RNR3-URA3* transcriptional fusion and spontaneous mutagenesis as means to identify mutants causing genome instability (Zhou and Elledge 1992). We sought to complement this approach using modern yeast genetic tools, incorporating the Rnr3 assay into a comprehensive genome-wide screen.

We generated a fluorescent reporter for *RNR3* expression compatible with reporter synthetic genetic array (R-SGA) technology, allowing us to systematically measure Rnr3 abundance across ~5200 yeast mutants (Kainth *et al.* 2009). We identified 150 mutants with increased Rnr3 abundance in the absence of exogenous perturbation and, in a second screen, 200 mutants with increased Rnr3 abundance in the presence of the DNA damaging agent MMS. We identified known repressors of *RNR3* transcription, such as *crt1∆*, validating our reporter system and saw high enrichment for processes known to impinge upon genome maintenance, such as DNA replication and repair, validating the use of Rnr3 abundance as an effective indicator of genome instability. Furthermore, by systematic comparison with existing genome instability datasets, we determined that 133 mutants, representing 130 different genes, 51% of which have human orthologs, are unique to our Rnr3 read-out of genome instability,

## Materials and Methods

### Rnr3 R-SGA

Two independent crosses of GBY691 (MATα *RNR3*-*GFP*::*HIS3MX leu2∆0 his3∆1 met15∆0 lyp1∆ can1pr*::*RPL39pr-tdTomato*::*CaURA3*::*can1∆*::*STE2pr-LEU2*) with the yeast nonessential deletion collection and a set of conditional temperature-sensitive alleles of essential genes (G. Tan and C. Boone, unpublished data) were performed following standard SGA procedures (Tong and Boone 2006). Final arrays were pinned in duplicate on either SD/MSG –his –leu –ura +200 mg/mL G418 (untreated) or YPD supplemented with 0.03% MMS, and grown for 20 hr before fluorescence scanning. Media was poured 48 hr before use, allowing for drying sufficient to prevent condensation on the fluorescence scanner. The Typhoon Trio Variable Mode Imager (GE Healthcare) was used to acquire GFP (488-nm laser, 520/40 BP emission filter) and tdTomato (532-nm laser, 610/30 BP emission filter) fluorescence values. For the essential temperature-sensitive mutants, all growth was conducted at 23°. After fluorescence imaging, colony size data were acquired by individually photographing plates with a Canon PowerShot G2 4.0 megapixel digital camera using Remote Capture software. Data analysis followed essentially what is described in Kainth *et al.* (2009), with small variations. To summarize, background-subtracted green fluorescent protein (GFP) and tdTomato intensities were computed for each colony from .GEL images using GenePix Pro version 3.0 software. Colony size information was calculated from individual photographs using Qt ColonyImager software, version 1.0.1. Border colonies, small colonies (colony area < 500 pixels), and colonies with aberrantly low tdTomato values (bottom 0.05%) were removed before further analysis. log_2_(Rnr3-GFP/tdTomato) values were calculated and LOESS normalized for each replicate experiment. Using the log_2_(Rnr3-GFP/tdTomato) ratio as a metric for Rnr3 abundance has the advantage that dividing by tdTomato corrects for any colony size-dependent intensity effects. Finally, normalized log_2_(Rnr3-GFP/tdTomato) values were averaged across all replicate experiments and a Z-score calculated (Supporting Information, Table S1 and Table S2; Raw R-SGA data, Supporting Information, File S1). All analysis was performed in R.

### Gene Ontology (GO) enrichment analysis

The GO Annotations Database was downloaded on September 23, 2014 (http://geneontology.org/page/download-annotations). A hypergeometric test was used to compute *P*-values for all GO terms within the biological process, molecular function and cellular component ontologies. GO terms with greater than 2000 associated genes were omitted as they are often too general to be informative. Similarly, for the biological process ontology, GO terms with less than 10 associated genes were omitted to remove very specific and largely redundant terms. *P*-values were adjusted for multiple hypothesis testing using the Benjamini-Hochberg method with a cut-off of FDR < 0.05.

### Homology analysis

The inParanoid8 database for *Saccharomyces cerevisiae* to *Homo sapiens* homology was downloaded (http://inparanoid.sbc.su.se/cgi-bin/e.cgi) to determine yeast-human homology relationships. The set of human cancer genes casually mutated in cancer were downloaded from the Cancer Gene Consensus (http://cancer.sanger.ac.uk/cancergenome/projects/census/).

## Results

### A genome-wide screen for mutants with increased genome instability using Rnr3 abundance

*RNR3* exhibits a strong and specific induction in response to genome instability in *S. cerevisiae*. We sought to adapt *RNR3* expression into a reporter for genome instability that would be amenable to a genome-wide mutant screen. To this end, we adopted the R-SGA methodology (Kainth *et al.* 2009), which allows for high-throughput acquisition of the abundance of a protein of interest in the context of a yeast mutant collection. We used R-SGA to query Rnr3 abundance across the set of yeast nonessential deletion mutants, containing ~4200 open-reading frame deletions (Giaever *et al.* 2002), as well as a set of ~1000 *kanMX*-marked, temperature-sensitive alleles that cover ~600 essential genes (G. Tan and C. Boone, unpublished data).

**Figure 1:**
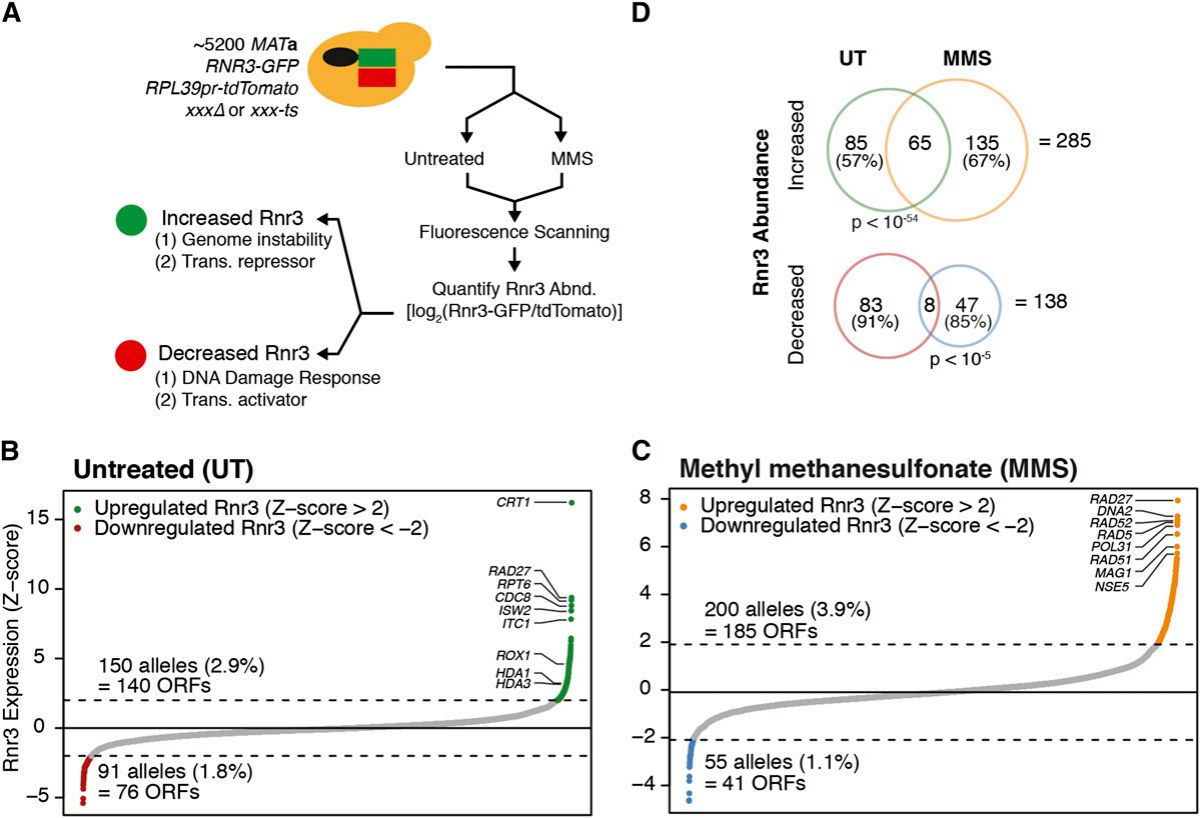
Assaying Rnr3 abundance across ~5200 yeast mutants, untreated (UT) and in methylmethane sulfonate (MMS). (A) Schematic of the Rnr3 R-SGA screening methodology. ~5200 yeast mutant strains containing *RNR3-GFP* and *RPL39pr-tdTomato* were either untreated or treated with MMS before fluorescence scanning and Rnr3 abundance quantification. Potential cellular functions of mutants with increased or decreased Rnr3 are described. (B) Distribution of Rnr3 abundances across all mutants screened in UT. (C) Distribution of Rnr3 abundance in MMS. (D) Overlap between UT and MMS screens for mutants with increased and decreased Rnr3 abundance. Both overlaps are significant. Total number of mutants unique to each condition is shown as a percentage, as well as the total number of mutants with increased and decreased Rnr3 across both conditions.

To conduct R-SGA, a strain containing endogenous *RNR3* C-terminally tagged with GFP and expressing tdTomato constitutively from the *RPL39* promoter was mated with both the non-essential gene deletion and conditional temperature-sensitive (TS) essential gene mutant collections using the SGA methodology (Tong and Boone 2006). The result was a collection of strains harboring *RNR3-GFP*, *RPL39pr-tdTomato*, and either a unique nonessential gene deletion (*xxxΔ*) or a TS essential gene allele (*xxx-TS*). Using a fluorescence scanner, we assayed the GFP and tdTomato intensities across the mutant strains arrayed as whole colonies on agar plates, and then we calculated a normalized log_2_(GFP/tdTomato) ratio, which is indicative of Rnr3 abundance. The screen was conducted in quadruplicate and on both untreated (UT) media and media containing the DNA-damaging agent MMS (Figure 1A). The average correlation across replicate screens was R = 0.73 and R = 0.95 for the nonessential deletion collection and temperature-sensitive collection, respectively. The correlations compare favorably with previous R-SGA screens, where an average of R = 0.77 was observed across 27 screens (Kainth *et al.* 2009).

We hypothesized that mutants causing increased *RNR3* expression in the absence of exogenous perturbation (untreated conditions) would unveil two classes of genes: (1) those directly regulating *RNR3* transcription and (2) those involved in the maintenance of genome stability (Figure 1A). By further performing the Rnr3 R-SGA screen in the presence of MMS (an exogenous source of DNA damage), we aimed to isolate genome maintenance factors that function specifically in tolerance of MMS damage.

We used a Z-score based threshold to identify mutants with the greatest change in Rnr3 abundance, defining those mutants with Z > 2 (corresponding to an Rnr3 abundance two standard deviations above the mean) as having increased Rnr3 and those with Z < −2 as having decreased Rnr3. Using these thresholds, we identified 150 mutants (2.9% of those screened) with increased Rnr3 abundance in untreated conditions and 91 with decreased Rnr3 abundance (Figure 1B, and Table 1, Table S3, and Table S4). In MMS, we identified 200 mutants (3.9%) with increased Rnr3 and 55 mutants with decreased Rnr3 (Figure 1C, Table 1, Table S5, and Table S6). A larger fraction of mutants with increased Rnr3 abundance were nonessential (~66% for UT and MMS); however, a greater relative proportion of all essential mutants screened had increased Rnr3 abundance (*e.g.*, ~34% of the 150 mutants with increased Rnr3 in UT are essential, although essential genes comprise only ~19% of the mutants screened). A tendency for mutation of essential genes to more readily cause genome instability is consistent with a previous study that similarly used both essential and nonessential mutant collections (Stirling *et al.* 2011).

**Table 1:** Evolutionary conservation of putative novel genome instability genes

	Untreated			MMS		
	Screened	Increased Rnr3	Increased Rnr3 and Novel	Screened	Increased Rnr3	Increased Rnr3 and Novel
Genes	5143	150	66	5121	200	85
Orthologs	1989 (39%)	78 (52%)	33 (50%)	1973 (39%)	106 (53%)	44 (52%)
γH2AX	356 (7%)	11 (7%)	3 (5%)	352 (7%)	17 (8.5%)	6 (7%)
CC	67 (1.3%)	3 (2%)	1 (1.5%)	67 (1.3%)	5 (2.5%)	3 (3.5%)

Gene mutants with an Rnr3 abundance Z-score >2 are indicated. Putative novel genome instability genes are defined in Figure 4. Orthologs were identified by the inParanoid algorithm. Genes with increased gH2AX foci following depletion were defined by Paulsen *et al.*, 2009. Genes included in the Cancer Census are indicated by CC. MMS, methylmethane sulfonate.

Altogether, we identified a total of 285 mutants that cause increased Rnr3 abundance and 138 mutants with decreased Rnr3 abundance. Both the UT and MMS screen shared a significant number of mutants with increased and decreased Rnr3 levels. Nevertheless, we identified a large number of mutants unique to each condition (Figure 1D). Indeed, MMS treatment resulted in the identification of an additional 135 mutants with increased and 47 mutants with decreased Rnr3, indicating a change in the complement of genes that determine Rnr3 abundance when DNA damage is present.

### Mutants with increased Rnr3 abundance

We first focused our attention on those mutants that exhibited an increase in Rnr3 abundance, which we expected to fall into two distinct functional classes: (1) those directly regulating *RNR3* transcription and (2) those involved in the maintenance of genome instability.

In a normal cell cycle, *RNR3* expression is inhibited by the transcriptional repressor Crt1/Rfx1, which binds to three DREs located upstream *RNR3*′*s* promoter (Huang *et al.* 1998). Consistent with this, a *CRT1* deletion mutant had by far the greatest abundance of Rnr3 in our R-SGA assay conducted in untreated conditions (Figure 1B). In addition, several other genes with established connections to *RNR* transcriptional repression, including *ISW2*, *HDA1*, and *ROX1* (Klinkenberg *et al.* 2006; Sharma *et al.* 2007; Zhang and Reese 2004), possessed an increased Rnr3 abundance upon deletion (Table S3). Our data also implicate *ITC1*, a binding partner of *ISW2* (Goldmark *et al.* 2000), and *HDA3*, which forms a complex with *HDA1* (Carmen *et al.* 1996), in the transcriptional repression of Rnr3, although this has not been explicitly demonstrated previously. For both complexes, Rnr3 induction upon deletion of either of the two constituent members was highly similar, suggesting that R-SGA can yield precise quantitative data on protein abundance. Together, these results confirm that the R-SGA methodology identifies known regulators of Rnr3 transcription.

Next, we sought to establish whether Rnr3 R-SGA could recover mutants belonging to our second expected class of hits: genes functioning to maintain genome stability. To take a broad and unbiased approach, we used the GO database, a curated set of terms describing known properties of the protein product of a gene. First, we examined the localization of genes whose mutation causes increased Rnr3 abundance. GO analysis using the cellular component ontology indicated that mutants with increased Rnr3 abundance are significantly enriched for genes that reside within the nucleus (Table S7). In fact, 79 of 140 genes with increased Rnr3 abundance have a nuclear localization, suggesting a direct role in genome maintenance. Analysis of the molecular function ontology supported the idea that many of the proteins with elevated Rnr3 abundance could directly influence genome stability. Namely, 31 genes possess direct DNA binding activity and 12 interact with chromatin (Table S7). Moreover, several genes contain domains that function to modify DNA topology, such as those possessing “DNA-dependent ATPase activity” (GO:0004003) and “helicase activity” (GO:0004386).

**Figure 2:**
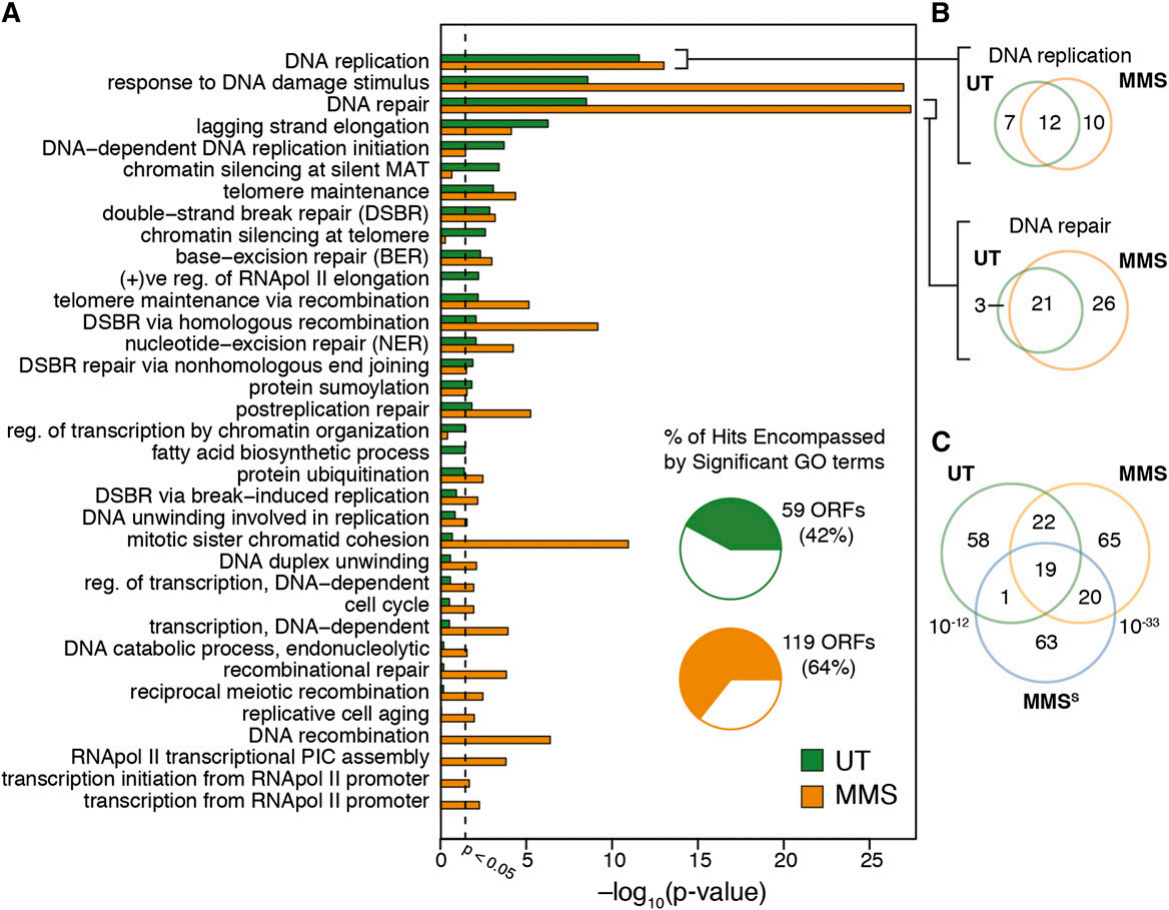
Significant biological processes present among mutants with increased Rnr3 abundance. (A) Breakdown of all biological process Gene Ontology (GO) terms significantly enriched among genes with increased Rnr3 in either the untreated (UT; green) or methylmethane sulfonate (MMS; yellow) screen. Terms are ordered from top by significance in the UT screen. Pie charts present the fraction of genes with increased Rnr3 annotated with at least one significant GO term. (B) Overlap of genes within the biological processes “DNA replication” (GO:0006260) and “DNA repair” (GO:0006281) between UT and MMS. (C) Overlap of nonessential genes with increased Rnr3 abundance with a set of nonessential mutants identified as MMS sensitive (MMS^S^). Both screens overlap significantly, but MMS has almost twice as many shared genes.

Finally, we examined the biological processes enriched among mutants with increased Rnr3 abundance in untreated conditions (Figure 2A). These terms overwhelmingly reflected processes that impinge upon genome stability. “DNA replication” (GO:0006260) was the most significantly enriched term, consistent with this biological process posing the greatest risk to genome stability in an unperturbed cell. Functionally similar GO terms “DNA repair” (GO:0006281) and “response to DNA damage stimulus” (GO:00060974) also were highly enriched. In aggregate, the 20 significantly enriched GO biological process terms included 59 unique mutants, covering 42% of all genes with increased Rnr3 abundance.

**Figure 3:**
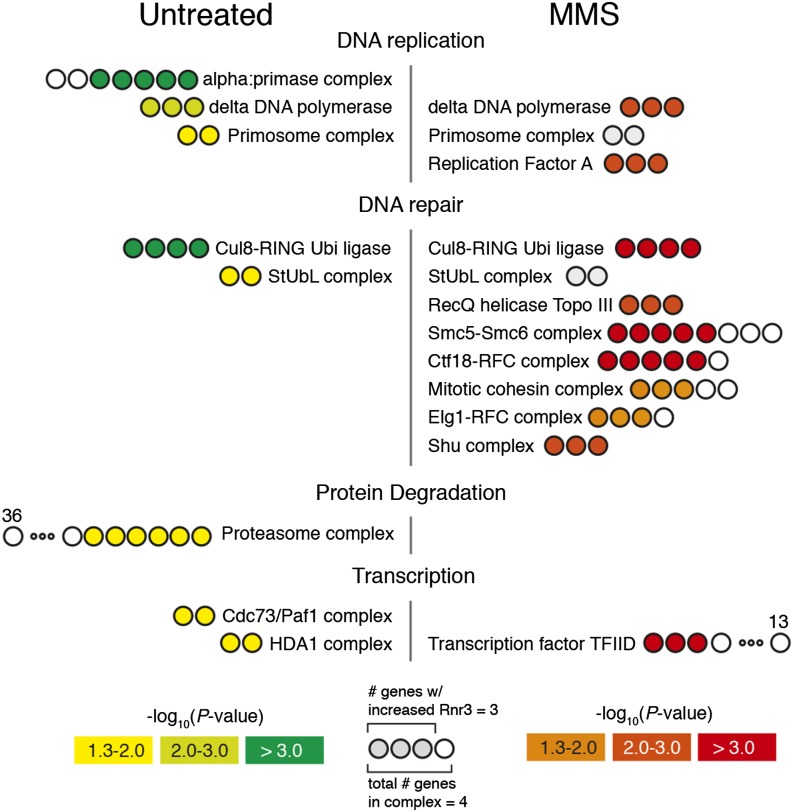
Significant protein complexes present among mutants with increased Rnr3 abundance. Protein complexes with significant enrichment in either the untreated or MMS screen are shown. Number of circles corresponds to the number of genes within the complex, filled circles indicates the number of those genes with increased Rnr3 abundance. Color corresponds to the significance of complex enrichment. Two complexes from the MMS screen with insignificant enrichment *P*-values are indicated in gray, as both their constituent genes were identified [StUbL (*i.e.*, SUMO-targeted Ubiquitin ligase) complex and Primosome complex]. Complexes were derived from the GO cellular component term set (Table S7 and Table S8). MMS, methylmethane sulfonate; GO, Gene Ontology.

In addition to uncovering many biological processes associated with genome instability, we also identified several protein complexes associated with the maintenance of genome stability (Figure 3). Interestingly, two of these complexes, the Cul8-RING Ubiquitin ligase complex and the StUbL (*i.e.*, SUMO-targeted Ubiquitin ligase) complex are related to ubiquitin transactions. All three members of lagging strand polymerase, DNA polymerase δ (*POL3*, *POL31*, *POL32*), were recovered, as well as both core members of DNA primase *(PRI1*, *PRI2)*.

### Mutants with increased Rnr3 abundance in MMS

We sought to expand our effort to understand genome stability to conditions of induced DNA damage. To this end, we repeated the Rnr3 R-SGA screen in the context of the carcinogen and DNA damaging agent MMS. MMS is a monofunctional DNA alkylating agent that primarily methylates DNA on *N^7^*-deoxyguanine and *N^3^*-deoxyadenine (Beranek 1990; Lawley 1989). The *N^3^*-methyladenine lesion in particular is thought to impede replication fork progression, which can lead to replication fork collapse (Beranek 1990). We reasoned that mutants with a greater increase in Rnr3 abundance in the presence of MMS would have functions specific to repair of MMS damage.

Results from the MMS Rnr3 R-SGA screen presented some commonalities with the UT Rnr3 R-SGA screen. For example, mutants with increased Rnr3 abundance in MMS were more likely to reside in the nucleus and have biochemical activities relevant to DNA maintenance (Table S8). Once again, “DNA replication” (GO:0006260), “DNA repair” (GO:0006821), and “response to DNA damage stimulus” (GO:0006974) were the three most significantly enriched GO biological process terms (Figure 2A). However, nearly double the number of genes associated with and the DDR were identified in the presence of MMS. In fact, the MMS screen identified nearly all the DNA repair annotated genes with increased Rnr3 abundance in UT conditions, plus an additional 26 DNA repair annotated genes (Figure 2B).

We wanted to assess the degree to which treatment with MMS resulted in the identification of mutants associated specifically with MMS repair. The *N^3^*-methyladenine lesions produced by MMS treatment are addressed through three mechanisms of repair: (1) base excision repair, (2) recombinational repair and (3) postreplication repair (Xiao *et al.* 1996). “Base-excision repair” (GO:0006284) and “postreplication repair” (GO:0006301) were significantly enriched terms in both UT and MMS screens; however, more BER and PRR genes were identified in the presence of MMS (6/15 BER genes in MMS *vs.* 5/15 in UT; 7/11 PRR genes in MMS *vs.* 4/11 in UT; Figure 2A). Notably, the base-excision repair gene *MAG1* (*i.e.*, 3-MethylAdenine DNA Glycosylase), the deletion of which increases the expression of *RNR3* in MMS (Jia and Xiao 2003), was identified specifically in the MMS Rnr3 R-SGA screen. The greater enrichment of postreplication repair was driven in part by identification of the entire E3 ubiquitin ligase complex containing *RAD5*, *UBC13*, and *MMS2*, which poly-ubquitylates proliferating cell nuclear antigen in an early step of error-free postreplication repair (Hoege *et al.* 2002). The final mechanism used in repair of MMS damage, “recombinational repair” (GO:0000725), was only enriched in the MMS screen (Figure 2A). Here, we identified all members of the Shu complex (*CSM2*, *PSY3*, *SHU1*), which mediates recombinational repair of stalled replication forks (Figure 3) (Ball *et al.* 2009). Other mutants found specifically in MMS were *MPH1*, which is thought to drive regression of stalled replication forks, and *MMS4* and *MUS81*, which play an important role in processing of stalled replication forks for homologous recombination (Choi *et al.* 2010; Osman and Whitby 2007). Finally, we noted a prominent difference between the two screens with regards to the process “mitotic sister chromatid cohesion” (GO:0007064), which was highly enriched only in the presence of MMS (Figure 2A).

As further evidence of the ability of our Rnr3 R-SGA screen to capture mutants involved in MMS repair, we compared mutants from both UT and MMS screens with a set of non-essential deletion mutants previously identified as being MMS sensitive (Figure 2C) (Chang *et al.* 2002). Both the UT and MMS Rnr3 R-SGA screen significantly overlapped with the MMS sensitive dataset. However, the MMS Rnr3 R-SGA screen had twice as many mutants in common with the MMS sensitivity screen as did the UT Rnr3 R-SGA screen.

### Comparison with previous genome instability screens

**Figure 4:**
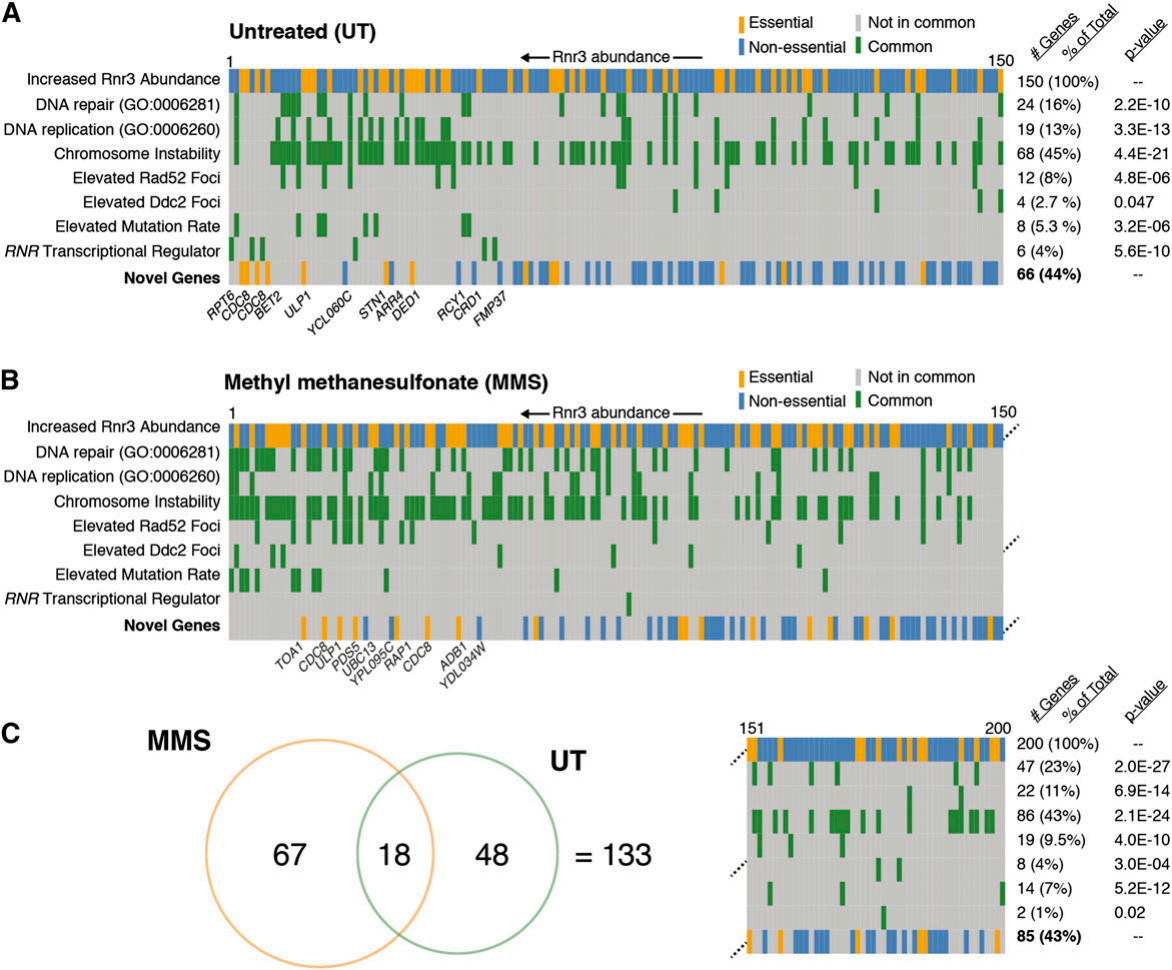
Systematic comparison with existing genome instability screens and genome maintenance-related GO terms. (A) Putative novel genome maintenance genes are identified by comparative gene subtraction. The 150 mutants with increased Rnr3 abundance in UT conditions are represented in each row, in order of decreasing Rnr3 abundance. In the top row, yellow rectangles represent essential and blue rectangles represent nonessential mutants. In the following seven rows, mutants are colored green if they are present in the data set listed at left, and gray otherwise. In the bottom row, all previously identified mutants are removed, leaving the putative novel mutants. Note that the majority of novel mutants cluster toward the right, indicating they have a lower Rnr3 abundance. The table on the right gives the number of genes with increased Rnr3 present in each data set, as well as the significance of the overlap between the datasets. (B) Same as in (A), but for the MMS screen. (C) Overlap between the putative novel genome maintenance genes identified in UT and MMS screens. A total of 133 novel mutants are identified. GO, Gene Ontology MMS, methylmethane sulfonate; UT, untreated.

Several genome instability studies have been conducted in *S. cerevisiae* (Alvaro *et al.* 2007; Alver *et al.* 2013; Andersen *et al.* 2008; Askree *et al.* 2004; Cheng *et al.* 2012; Huang *et al.* 2003; Scholes *et al.* 2001; Smith *et al.* 2004; Stirling *et al.* 2011). Many of these studies have made use of the same collection of nonessential deletion mutants and more recently the use of hypomorphic or temperature-sensitive mutant collections similar to the one we used has been reported (Cheng *et al.* 2012; Stirling *et al.* 2011). We used this existing pool of data to conduct a thorough assessment of whether any of the 285 mutants with increased Rnr3 abundance could potentially represent novel genome maintenance genes. We focused on conducting a comparison to studies that directly assayed genome instability, as drug sensitivity and global protein localization studies can capture effects additional to genome maintenance (Tkach *et al.* 2012). To this end, we chose four major and orthogonal genome instability screens done in yeast to cross reference with our dataset: (1) 33 nonessential mutants possessing an elevated mutation rate (Huang *et al.* 2003), (2) 87 essential mutants with increased Ddc2 foci (Cheng *et al.* 2012), an indication of exposed ssDNA tracts, (3) 89 nonessential mutants with elevated Rad52 foci (Alvaro *et al.* 2007), an indication of double strand DNA breaks, and (4) 692 essential and nonessential mutants with chromosome instability (Stirling *et al.* 2011), roughly defined as increased loss of genetic material, as assessed by several different assays. In addition, we collected genes with the GO annotations “DNA repair” (GO:0006281) and “DNA replication” (GO:0006260), to capture mutants with a known association to these processes that haven’t been captured in high-throughput genome instability studies. We systematically annotated each mutant with increased Rnr3 abundance for its presence in any of the four genome instability datasets or two GO terms, and also annotated known transcriptional regulators of *RNR* (Figure 4). 

From the pool of mutants with increased Rnr3 in untreated conditions, 84 mutants could be assigned to at least one of the above annotations leaving 66 mutants (44%) as potentially novel genome maintenance genes (Table S9). Similarly, 115 mutants with increased Rnr3 abundance in MMS had at least one genome instability annotation, leaving 85 (43%) mutants unannotated (Table S10). For both the UT and MMS screen, putative novel mutants had a tendency to be ranked lower than those associated with existing genome instability datasets. Overlap of the two sets of putative novel mutants revealed 18 shared mutants, resulting in an aggregate of 133 novel mutants identified (Figure 4C). Among these genes, GO enrichment produced no significantly enriched terms, suggesting that the putative novel mutants are not functionally cohesive and derive from a wide array of biological processes. Some of the putative novel mutants have inferable connections to genome maintenance, despite not being identified in previous genome instability screens. For example, we identified two alleles of *CDC8* (*cdc8-1 and cdc8-2*), which is involved in dTTP synthesis (Jong *et al.* 1984; Sclafani and Fangman 1984), and therefore likely causes genome instability by affecting DNA replication (Hartwell 1973). Others are less easily linked to genome maintenance, such as *DED1* (again supported by two alleles, *ded1-95* and *ded1-199*), an ATP-dependent RNA helicase important for translation initiation (Chuang *et al.* 1997; Iost *et al.* 1999).

### Evolutionary conservation of mutants with increased Rnr3 abundance

We used the ortholog cluster database generated by the inParanoid algorithm to evaluate the extent to which genes with increased Rnr3 abundance are conserved from yeast to human (Sonnhammer and Ostlund 2014). The core of the inParanoid algorithm is a reciprocal best-match BLAST search between proteomes of two species of interest, which is used to identify ortholog pairs. In total, the inParanoid algorithm assigns 2386 yeast genes a human ortholog, corresponding to 36% of the yeast genome. We identified 78 (52%) yeast genes with human orthologs among the set of genes with increased Rnr3 abundance in untreated conditions (Table S11). Similarly, 106 (53%) of the mutants with increased Rnr3 in MMS had a human ortholog (Table S12). Interestingly, considering only our putative novel set of mutants has little effect on the fraction of genes conserved: 33 (50%) novel UT and 44 (52%) novel MMS mutants have human orthologs (Table 1).

We compared the human orthologs of our genes causing increased Rnr3 abundance to two additional datasets. The first was a set of human genes identified as having increased H2AX phosphorylation following small interfering RNA knock-down (Paulsen *et al.* 2009). H2AX is phosphorylated at residue Ser139 in response to double-strand breaks by DNA damage checkpoint kinases, and serves as an indicator of genome instability in human cells (Paulsen *et al.* 2009). We found 11 and 17 of the mutants with increased Rnr3 abundance in UT and MMS had a human homolog with increased H2AX phosphorylation (Table 1, Table S11, and Table S12), suggesting their role in genome maintenance is conserved. Of these, eight belong to our set of putative novel mutants. In addition, we asked whether any of the human homologs of yeast mutants with increased Rnr3 were present in the Cancer Gene Census, which is a list of genes with mutations that have been causally linked to oncogenesis (Forbes *et al.* 2014; Futreal *et al.* 2004). In doing so, we identified a total of seven mutants present in the cancer census that have increased Rnr3 abundance in yeast, four of which were associated with our putative novel set.

## Discussion

We have complemented a previous version of the Rnr3 assay (Zhou and Elledge 1992) by using yeast functional genomics reagents and methodologies. Instead of screening mutants generated via spontaneous mutagenesis, we us the nonessential gene deletion collection and conditional temperature-sensitive gene mutant collection. We introduce an Rnr3 reporter into both of these mutant collections using R-SGA. The resultant assay is an extremely high-throughput method to screen for genome instability: from start to finish it entails only an SGA procedure followed by ~1 hr of fluorescence scanning of whole colonies arrayed on agar plates (for the ~4800 mutants in the yeast nonessential deletion collection). Moreover, the method is compatible with drug treatment, insofar as the drug of interest is soluble in agar plates.

The Zhou and Elledge Rnr3 assay identified nine complementation groups (*CRT1* to *CRT9*) exhibiting increased Rnr3 (Zhou and Elledge 1992). Of these, the associated gene for all but *CRT2* is known. These genes are the transcriptional regulators *CRT1/RFX1*, *SSN6*, and *TUP1*; the RNR subunits *RNR1*, *RNR2*,and *RNR4*; and *CDC21*. Our R-SGA Rnr3 screen robustly identified *CRT1*/*RFX1*. Alleles of *SSN6*, *TUP1*, *RNR1*, *RNR2*, and *RNR4* were not present in the mutant collections screened. An allele of *CDC21* was screened in our R-SGA assay and did not have increased Rnr3 abundance, although another gene in the *de novo* pyrimidine synthesis pathway, *CDC8*, was identified.

Although here we focused on mutants causing increased Rnr3, we also identified 138 mutants with decreased Rnr3 (Table S4 and Table S6). Interestingly, in the MMS Rnr3 R-SGA screen, we noted that mutants with decreased Rnr3 abundance were significantly enriched for genes associated with the DDR pathway (data not shown). This result makes sense, as Rnr3 induction in the presence of damage depends on an intact DDR and suggests a further class of genes may be identified through screening Rnr3 abundance in the presence of damage.

A number of genome instability screens have been previously conducted in *S. cerevisiae*. This existing set of screens provides a valuable resource for comparative analysis. For example, after systematic comparison of our Rnr3 R-SGA dataset with four major, orthogonal genome instability studies, as well as GO terms associated with genome maintenance, we conclude that 45% of the 285 mutants we identified have no previous connection to maintenance of the genome. We attribute this substantial number of novel mutants to Rnr3 providing a different measure of genome instability relative to earlier screens. Existing genome instability screens can be roughly divided into two classes, based on the nature of their read-out: (1) those that directly assay alterations in the genetic material (such as mutation of the canavinine reporter, chromosome loss, loss of the MAT locus, telomere length, etc.) and (2) those that infer the presence of DNA damage through monitoring the formation of “damage foci” by fluorescence microscopy. Distinct from these approaches, the Rnr3 R-SGA identifies mutants that cause induction of the DDR.

We sought to assess the extent to which insights in yeast can be transferred to human and, in particular, to our understanding of genome instability in cancer. Approximately 52% of the mutants exhibiting increased Rnr3 abundance have human homologs, compared with 36% of all yeast genes. The enrichment for homologs is partly reflective of the fact that core genome maintenance processes, such as DNA replication, are highly conserved (75% of genes associated with the GO term “DNA replication” have human homologs). However, even in considering only the putative novel set of mutants, which lack known connections to replication or repair, we still note that 51% have human homologs. Furthermore, in about 14% of cases, we are able to provide evidence of conservation of the genome instability phenotype from yeast to human, simply by comparing to an existing genome instability screen. Although neither the UT nor the MMS Rnr3 R-SGA screen was significantly enriched for human genome instability genes, we note that, just as in comparisons between genome instability screens in yeast, differences in read-out between human and yeast screens can produce nonoverlapping results. Other factors likely frustrate such a direct approach to assessing phenotypic conservation, such as extent of inactivation of the gene (knock-down *vs.* deletion and hypomorph) and cell-type specific effects in human. Lastly, we recovered a handful of genes with human homologs that are part of the Cancer Gene Consensus, demonstrating that yeast genome instability studies can be used to provide disease relevant functional annotations to human cancer genes.

More than half of all genes causally implicated in cancer are oncogenes (Vogelstein *et al.* 2013). Indeed, gene duplication or hyperactivity is a frequent occurrence in cancer, yet the extent to which this causes genome instability is unknown. Gene overexpression collections now exist in yeast (Douglas *et al.* 2012; Hu *et al.* 2007; Sopko *et al.* 2006), and can be readily combined with the Rnr3 R-SGA method, providing insights into the relationship between oncogenes and genome instability.

## References

[bib1] Agarwal R. Tang Z. Yu H. Cohen-Fix O., 2003 Two distinct pathways for inhibiting pds1 ubiquitination in response to DNA damage. J. Biol. Chem. 278: 45027–45033. 12947083 10.1074/jbc.M306783200

[bib2] Aguilera A. Gomez-Gonzalez B., 2008 Genome instability: a mechanistic view of its causes and consequences. Nat. Rev. Genet. 9: 204–217. 18227811 10.1038/nrg2268

[bib3] Alvaro D. Lisby M. Rothstein R., 2007 Genome-wide analysis of Rad52 foci reveals diverse mechanisms impacting recombination. PLoS Genet. 3: e228. 18085829 10.1371/journal.pgen.0030228 PMC2134942

[bib4] Alver B. Jauert P. A. Brosnan L. O’Hehir M. Vandersluis B., 2013 A whole genome screen for minisatellite stability genes in stationary phase yeast cells. G3 (Bethesda) 3: 741–756. 10.1534/g3.112.005397 PMC3618361 23550123

[bib5] Andersen M. P. Nelson Z. W. Hetrick E. D. Gottschling D. E., 2008 A genetic screen for increased loss of heterozygosity in *Saccharomyces cerevisiae*. Genetics 179: 1179–1195. 18562670 10.1534/genetics.108.089250 PMC2475725

[bib6] Askree S. H. Yehuda T. Smolikov S. Gurevich R. Hawk J., 2004 A genome-wide screen for *Saccharomyces cerevisiae* deletion mutants that affect telomere length. Proc. Natl. Acad. Sci. USA 101: 8658–8663. 15161972 10.1073/pnas.0401263101 PMC423251

[bib7] Ball L. G. Zhang K. Cobb J. A. Boone C. Xiao W., 2009 The yeast Shu complex couples error-free post-replication repair to homologous recombination. Mol. Microbiol. 73: 89–102. 19496932 10.1111/j.1365-2958.2009.06748.x

[bib8] Beranek D. T., 1990 Distribution of methyl and ethyl adducts following alkylation with monofunctional alkylating agents. Mutat. Res. 231: 11–30. 2195323 10.1016/0027-5107(90)90173-2

[bib9] Carmen A. A. Rundlett S. E. Grunstein M., 1996 HDA1 and HDA3 are components of a yeast histone deacetylase (HDA) complex. J. Biol. Chem. 271: 15837–15844. 8663039 10.1074/jbc.271.26.15837

[bib10] Chabes A. Georgieva B. Domkin V. Zhao X. Rothstein R., 2003 Survival of DNA damage in yeast directly depends on increased dNTP levels allowed by relaxed feedback inhibition of ribonucleotide reductase. Cell 112: 391–401. 12581528 10.1016/s0092-8674(03)00075-8

[bib11] Chang M. Bellaoui M. Boone C. Brown G. W., 2002 A genome-wide screen for methyl methanesulfonate-sensitive mutants reveals genes required for S phase progression in the presence of DNA damage. Proc. Natl. Acad. Sci. USA 99: 16934–16939. 12482937 10.1073/pnas.262669299 PMC139247

[bib12] Cheng E. Vaisica J. A. Ou J. Baryshnikova A. Lu Y., 2012 Genome rearrangements caused by depletion of essential DNA replication proteins in *Saccharomyces cerevisiae*. Genetics 192: 147–160. 22673806 10.1534/genetics.112.141051 PMC3430531

[bib13] Choi K. Szakal B. Chen Y. H. Branzei D. Zhao X., 2010 The Smc5/6 complex and Esc2 influence multiple replication-associated recombination processes in *Saccharomyces cerevisiae*. Mol. Biol. Cell 21: 2306–2314. 20444977 10.1091/mbc.E10-01-0050 PMC2893993

[bib14] Chuang R.-Y. Weaver P. L. Liu Z. Chang T.-H., 1997 Requirement of the DEAD-Box protein ded1p for messenger RNA translation. Science 275: 1468–1471. 9045610 10.1126/science.275.5305.1468

[bib15] Davidson M. B. Katou Y. Keszthelyi A. Sing T. L. Xia T., 2012 Endogenous DNA replication stress results in expansion of dNTP pools and a mutator phenotype. EMBO J. 31: 895–907. 22234187 10.1038/emboj.2011.485 PMC3280564

[bib16] Douglas A.C. Smith A.M. Sharifpoor S. Yan Z. Durbic T., 2012 Functional analysis with a barcoder yeast gene overexpression system. G3 (Bethesda) 2: 1279–1289. 23050238 10.1534/g3.112.003400 PMC3464120

[bib17] Elledge S. J. Zhou Z. Allen J. B. Navas T. A., 1993 DNA damage and cell cycle regulation of ribonucleotide reductase. BioEssays 15: 333–339. 8343143 10.1002/bies.950150507

[bib18] Falck J. Coates J. Jackson S. P., 2005 Conserved modes of recruitment of ATM, ATR and DNA-PKcs to sites of DNA damage. Nature 434: 605–611. 15758953 10.1038/nature03442

[bib19] Forbes S. A. Beare D. Gunasekaran P. Leung K. Bindal N., 2015 COSMIC: exploring the world’s knowledge of somatic mutations in human cancer. Nucleic Acids Res. 43 **(**Database Issue**):** D805–D811. 25355519 10.1093/nar/gku1075 PMC4383913

[bib20] Futreal P. A. Coin L. Marshall M. Down T. Hubbard T., 2004 A census of human cancer genes. Nat. Rev. Cancer 4: 177–183. 14993899 10.1038/nrc1299 PMC2665285

[bib21] Giaever G. Chu A. M. Ni L. Connelly C. Riles L., 2002 Functional profiling of the *Saccharomyces cerevisiae* genome. Nature 418: 387–391. 12140549 10.1038/nature00935

[bib22] Glickman M. S. Sawyers C. L., 2012 Converting cancer therapies into cures: lessons from infectious diseases. Cell 148: 1089–1098. 22424221 10.1016/j.cell.2012.02.015 PMC3465702

[bib23] Goldmark J. P. Fazzio T. G. Estep P. W. Church G. M. Tsukiyama T., 2000 The Isw2 chromatin remodeling complex represses early meiotic genes upon recruitment by Ume6p. Cell 103: 423–433. 11081629 10.1016/s0092-8674(00)00134-3

[bib24] Harrison J. C. Haber J. E., 2006 Surviving the breakup: the DNA damage checkpoint. Annu. Rev. Genet. 40: 209–235. 16805667 10.1146/annurev.genet.40.051206.105231

[bib25] Hartwell L. H., 1973 Three additional genes required for deoxyribonucleic acid synthesis in *Saccharomyces cerevisiae*. J. Bacteriol. 115: 966–974. 4580573 10.1128/jb.115.3.966-974.1973 PMC246343

[bib26] Hoege C. Pfander B. Moldovan G. L. Pyrowolakis G. Jentsch S., 2002 RAD6-dependent DNA repair is linked to modification of PCNA by ubiquitin and SUMO. Nature 419: 135–141. 12226657 10.1038/nature00991

[bib27] Hu Y. Rolfs A. Bhullar B. Murthy T. V. Zhu C., 2007 Approaching a complete repository of sequence-verified protein-encoding clones for *Saccharomyces cerevisiae*. Genome Res. 17: 536–543. 17322287 10.1101/gr.6037607 PMC1832101

[bib28] Huang M. Zhou Z. Elledge S. J., 1998 The DNA replication and damage checkpoint pathways induce transcription by inhibition of the Crt1 repressor. Cell 94: 595–605. 9741624 10.1016/s0092-8674(00)81601-3

[bib29] Huang M. E. Rio A. G. Nicolas A. Kolodner R. D., 2003 A genomewide screen in *Saccharomyces cerevisiae* for genes that suppress the accumulation of mutations. Proc. Natl. Acad. Sci. USA 100: 11529–11534. 12972632 10.1073/pnas.2035018100 PMC208792

[bib30] Iost I. Dreyfus M. Linder P., 1999 Ded1p, a DEAD-box protein required for translation initiation in *Saccharomyces cerevisiae*, is an RNA helicase. J. Biol. Chem. 274: 17677–17683. 10364207 10.1074/jbc.274.25.17677

[bib31] Jia X. Xiao W., 2003 Compromised DNA repair enhances sensitivity of the yeast RNR3-lacZ genotoxicity testing system. Toxicol. Sci. 75: 82–88. 12805645 10.1093/toxsci/kfg158

[bib32] Jong A. Y. Kuo C. L. Campbell J. L., 1984 The CDC8 gene of yeast encodes thymidylate kinase. J. Biol. Chem. 259: 11052–11059. 6088527

[bib33] Kainth P. Sassi H. E. Pena-Castillo L. Chua G. Hughes T. R., 2009 Comprehensive genetic analysis of transcription factor pathways using a dual reporter gene system in budding yeast. Methods 48: 258–264. 19269327 10.1016/j.ymeth.2009.02.015

[bib34] Klinkenberg L. G. Webb T. Zitomer R. S., 2006 Synergy among differentially regulated repressors of the ribonucleotide diphosphate reductase genes of *Saccharomyces cerevisiae*. Eukaryot. Cell 5: 1007–1017. 16835445 10.1128/EC.00045-06 PMC1489293

[bib35] Lawley P. D., 1989 Mutagens as carcinogens: development of current concepts. Mutat. Res. 213: 3–25. 2664491 10.1016/0027-5107(89)90028-6

[bib36] Li B. Reese J. C., 2001 Ssn6-Tup1 regulates RNR3 by positioning nucleosomes and affecting the chromatin structure at the upstream repression sequence. J. Biol. Chem. 276: 33788–33797. 11448965 10.1074/jbc.M104220200

[bib37] Lopez-Mosqueda J. Maas N. L. Jonsson Z. O. Defazio-Eli L. G. Wohlschlegel J., 2010 Damage-induced phosphorylation of Sld3 is important to block late origin firing. Nature 467: 479–483. 20865002 10.1038/nature09377 PMC3393088

[bib38] Majka J. Niedziela-Majka A. Burgers P. M., 2006 The checkpoint clamp activates Mec1 kinase during initiation of the DNA damage checkpoint. Mol. Cell 24: 891–901. 17189191 10.1016/j.molcel.2006.11.027 PMC1850967

[bib39] Naiki T. Wakayama T. Nakada D. Matsumoto K. Sugimoto K., 2004 Association of Rad9 with double-strand breaks through a Mec1-dependent mechanism. Mol. Cell. Biol. 24: 3277–3285. 15060150 10.1128/MCB.24.8.3277-3285.2004 PMC381673

[bib40] Nakada D. Matsumoto K. Sugimoto K., 2003 ATM-related Tel1 associates with double-strand breaks through an Xrs2-dependent mechanism. Genes Dev. 17: 1957–1962. 12923051 10.1101/gad.1099003 PMC196250

[bib41] Navadgi-Patil V. M. Burgers P. M., 2009 The unstructured C-terminal tail of the 9–1−1 clamp subunit Ddc1 activates Mec1/ATR via two distinct mechanisms. Mol. Cell 36: 743–753. 20005839 10.1016/j.molcel.2009.10.014 PMC2796261

[bib42] Negrini S. Gorgoulis V. G. Halazonetis T. D., 2010 Genomic instability—an evolving hallmark of cancer. Nat. Rev. Mol. Cell Biol. 11: 220–228. 20177397 10.1038/nrm2858

[bib43] Osman F. Whitby M. C., 2007 Exploring the roles of Mus81-Eme1/Mms4 at perturbed replication forks. DNA Repair (Amst.) 6: 1004–1017. 17409028 10.1016/j.dnarep.2007.02.019

[bib44] Paulsen R. D. Soni D. V. Wollman R. Hahn A. T. Yee M. C., 2009 A genome-wide siRNA screen reveals diverse cellular processes and pathways that mediate genome stability. Mol. Cell 3: 228–239. 19647519 10.1016/j.molcel.2009.06.021 PMC2772893

[bib45] Putnam C. D. Jaehnig E. J. Kolodner R. D., 2009 Perspectives on the DNA damage and replication checkpoint responses in *Saccharomyces cerevisiae*. DNA Repair (Amst.) 8: 974–982. 19477695 10.1016/j.dnarep.2009.04.021 PMC2725198

[bib46] Rouse J. Jackson S. P., 2002 Lcd1p recruits Mec1p to DNA lesions in vitro and in vivo. Mol. Cell 9: 857–869. 11983176 10.1016/s1097-2765(02)00507-5

[bib47] Sanchez Y. Bachant J. Wang H. Hu F. Liu D., 1999 Control of the DNA damage checkpoint by chk1 and rad53 protein kinases through distinct mechanisms. Science 286: 1166–1171. 10550056 10.1126/science.286.5442.1166

[bib48] Sanvisens N. de Llanos R. Puig S., 2013 Function and regulation of yeast ribonucleotide reductase: cell cycle, genotoxic stress, and iron bioavailability. Biom. J. 36: 51–58. 10.4103/2319-4170.110398 23644233

[bib49] Scholes D. T. Banerjee M. Bowen B. Curcio M. J., 2001 Multiple regulators of Ty1 transposition in *Saccharomyces cerevisiae* have conserved roles in genome maintenance. Genetics 159: 1449–1465. 11779788 10.1093/genetics/159.4.1449 PMC1461915

[bib50] Schwartz M. F. Duong J. K. Sun Z. Morrow J. S. Pradhan D., 2002 Rad9 Phosphorylation sites couple Rad53 to the *Saccharomyces cerevisiae* DNA damage checkpoint. Mol. Cell 9: 1055–1065. 12049741 10.1016/s1097-2765(02)00532-4

[bib51] Sclafani R. A. Fangman W. L., 1984 Yeast gene CDC8 encodes thymidylate kinase and is complemented by herpes thymidine kinase gene TK. Proc. Natl. Acad. Sci. USA 81: 5821–5825. 6091111 10.1073/pnas.81.18.5821 PMC391803

[bib52] Sharma V. M. Tomar R. S. Dempsey A. E. Reese J. C., 2007 Histone deacetylases RPD3 and HOS2 regulate the transcriptional activation of DNA damage-inducible genes. Mol. Cell. Biol. 27: 3199–3210. 17296735 10.1128/MCB.02311-06 PMC1899941

[bib53] Smith S. Hwang J. Y. Banerjee S. Majeed A. Gupta A., 2004 Mutator genes for suppression of gross chromosomal rearrangements identified by a genome-wide screening in *Saccharomyces cerevisiae*. Proc. Natl. Acad. Sci. USA 101: 9039–9044. 15184655 10.1073/pnas.0403093101 PMC428469

[bib54] Sonnhammer E. L. Ostlund G., 2014 InParanoid 8: orthology analysis between 273 proteomes, mostly eukaryotic. Nucleic Acids Res. 43 **(**Database Issue**):** D234–D239. 25429972 10.1093/nar/gku1203 PMC4383983

[bib55] Sopko R. Huang D. Preston N. Chua G. Papp B., 2006 Mapping pathways and phenotypes by systematic gene overexpression. Mol. Cell 21: 319–330. 16455487 10.1016/j.molcel.2005.12.011

[bib56] Stirling P. C. Bloom M. S. Solanki-Patil T. Smith S. Sipahimalani P., 2011 The complete spectrum of yeast chromosome instability genes identifies candidate CIN cancer genes and functional roles for ASTRA complex components. PLoS Genet. 7: e1002057. 21552543 10.1371/journal.pgen.1002057 PMC3084213

[bib57] Storchova Z. Pellman D., 2004 From polyploidy to aneuploidy, genome instability and cancer. Nat. Rev. Mol. Cell Biol. 5: 45–54. 14708009 10.1038/nrm1276

[bib58] Sun Z. Hsiao J. Fay D. S. Stern D. F., 1998 Rad53 FHA domain associated with phosphorylated Rad9 in the DNA damage checkpoint. Science 281: 272–274. 9657725 10.1126/science.281.5374.272

[bib59] Sweeney F. D. Yang F. Chi A. Shabanowitz J. Hunt D. F., 2005 *Saccharomyces cerevisiae* Rad9 acts as a Mec1 adaptor to allow Rad53 activation. Curr. Biol. 15: 1364–1375. 16085488 10.1016/j.cub.2005.06.063

[bib60] Tang H. M. Siu K. L. Wong C. M. Jin D. Y., 2009 Loss of yeast peroxiredoxin Tsa1p induces genome instability through activation of the DNA damage checkpoint and elevation of dNTP levels. PLoS Genet. 5: e1000697. 19851444 10.1371/journal.pgen.1000697 PMC2758587

[bib61] Tkach J. M. Yimit A. Lee A. Y. Riffle M. Costanzo M., 2012 Dissecting DNA damage response pathways by analysing protein localization and abundance changes during DNA replication stress. Nat. Cell Biol. 14: 966–976. 22842922 10.1038/ncb2549 PMC3434236

[bib62] Tong A. H. Boone C., 2006 Synthetic genetic array analysis in Saccharomyces cerevisiae. Methods Mol. Biol. 313: 171–192. 16118434 10.1385/1-59259-958-3:171

[bib63] Tsaponina O. Barsoum E. Astrom S. U. Chabes A., 2011 Ixr1 is required for the expression of the ribonucleotide reductase Rnr1 and maintenance of dNTP pools. PLoS Genet. 7: e1002061. 21573136 10.1371/journal.pgen.1002061 PMC3088718

[bib64] Vogelstein B. Papadopoulos N. Velculescu V. E. Zhou S. Diaz L. A. Jr, 2013 Cancer genome landscapes. Science 339: 1546–1558. 23539594 10.1126/science.1235122 PMC3749880

[bib65] Xiao W. Chow B. L. Rathgeber L., 1996 The repair of DNA methylation damage in Saccharomyces cerevisiae. Curr. Genet. 30: 461–468. 8939806 10.1007/s002940050157

[bib66] Yu L. Pena Castillo L. Mnaimneh S. Hughes T. R. Brown G. W., 2006 A survey of essential gene function in the yeast cell division cycle. Mol. Biol. Cell 17: 4736–4747. 16943325 10.1091/mbc.E06-04-0368 PMC1635385

[bib67] Zegerman P. Diffley J. F. X., 2010 Checkpoint-dependent inhibition of DNA replication initiation by Sld3 and Dbf4 phosphorylation. Nature 467: 474–478. 20835227 10.1038/nature09373 PMC2948544

[bib68] Zhang Z. Reese J. C., 2004 Ssn6-Tup1 requires the ISW2 complex to position nucleosomes in *Saccharomyces cerevisiae*. EMBO J. 23: 2246–2257. 15116071 10.1038/sj.emboj.7600227 PMC419907

[bib69] Zhou Z. Elledge S. J., 1992 Isolation of crt mutants constitutive for transcription of the DNA damage inducible gene RNR3 in *Saccharomyces cerevisiae*. Genetics 131: 851–866. 1516817 10.1093/genetics/131.4.851 PMC1205097

[bib70] Zou L. Elledge S. J., 2003 Sensing DNA damage through ATRIP recognition of RPA-ssDNA complexes. Science 300: 1542–1548. 12791985 10.1126/science.1083430

